# Oxytocin enhances neural approach towards social and non-social stimuli of high personal relevance

**DOI:** 10.1038/s41598-021-02914-8

**Published:** 2021-12-08

**Authors:** Kaat Alaerts, Aymara Taillieu, Nicky Daniels, Javier R. Soriano, Jellina Prinsen

**Affiliations:** Department of Rehabilitation Sciences, Research Group for Neurorehabilitation, KU Leuven, Tervuursevest 101 box 1501, 3001 Leuven, Belgium

**Keywords:** Neuroscience, Psychology

## Abstract

Oxytocin (OT) plays a pivotal role in a variety of complex social behaviors by modulating approach-avoidance motivational tendencies, but recently, its social specificity has been challenged. Here, a randomized, double-blind, placebo-controlled study was conducted with forty young adult men, investigating the effect of a single-dose of OT (24 IU) on behavioral and neural approach-avoidance. Frontal alpha asymmetry, indexing neurophysiological approach-avoidance, was obtained from electroencephalographic recordings while participants were presented with a series of pictures, individually rated in terms of personal relevance (i.e., high versus low positive/negative emotional evocativeness) and categorized as social or non-social. Additionally, participants could prolong (approach) or shorten (avoid) the viewing-time of each picture, providing a measure of behavioral approach-avoidance. Intranasal OT enhanced both behavioral and neural approach (increased viewing-time), particularly towards negatively valenced pictures of both social and non-social nature, thus challenging the notion that OT’s effects are specific to social stimuli. Neurally, OT specifically amplified approach-related motivational salience of stimuli that were self-rated to have high personal relevance, but irrespective of their social nature or rated affective valence (positive/negative). Together, these findings provide support to the General Approach-Avoidance Hypothesis of OT, suggesting a role of OT in amplifying the motivational salience of environmental stimuli with high (personal) relevance, but irrespective of their social/non-social nature.

**Clinical Trial Number**: The study design was registered at ClinicalTrials.gov (NCT04443647; 23/06/2020; https://clinicaltrials.gov/ct2/show/NCT04443647).

## Introduction

The neuropeptide oxytocin (OT) plays an important role in a wide range of complex social behaviors. Related to its implicated role in promoting ‘pro-social’ behavior, intranasal administration of OT is increasingly considered as a potential treatment for a variety of neuropsychiatric disorders characterized by difficulties in the social domain, such as autism spectrum disorder (ASD). However, recent research has challenged prevailing theories on the pro-sociality of OT by demonstrating that—depending on context and/or person-dependent characteristics—OT may not uniformly induce ‘pro-social’, but also opposite or ‘anti-social’ effects^1^. For example, Shamay-Tsoory^2^, demonstrated that OT not only mediated positive social emotions and behaviors, but also negative anti-social behaviors such as envy and gloating^2^. Also De Dreu et al.^3^ showed that OT facilitates a "tend and defend" response, not only by promoting trust and cooperation among in-group members, but also by promoting defensive aggression towards out-group members^3^. The influential social salience account of OT was proposed to reconcile these observations, positing that—irrespective of valence—OT increases the salience and/or (central) processing of social cues, thereby resulting in a corresponding up-regulation of both positively and negatively valenced social emotions and behaviors (i.e., depending on the context of the presented social interaction)^2,4^. In contrast, other mechanistic accounts have been put forward, suggesting that OT’s central action can be understood in terms of its modulatory impact on approach-avoidance motivational tendencies. Within the social approach/avoidance framework proposed by Kemp and Guastella^5^, OT is suggested to induce a down-regulation of avoidance-related behavior and an up-regulation of approach-related behavior, by inducing a decrease in the experience of social threats and an increase in the recognition of social-reward-driven stimuli, respectively^5^.

More recently, also the ‘social’ specificity of OT’s action was challenged. In order to account for several observations of non-social effect of OT, such as OT-induced reduction of stress in non-social tasks in rodents^6^ or increased eye gaze towards both social and non-social stimuli in humans^7^, the social approach/avoidance account was further elaborated into the General Approach-Avoidance Hypothesis of OT (GAAO;^8^). Specifically, within the GAAO account^8^, OT is proposed to modulate approach/avoidance motivational tendencies and behaviors by impacting the meso-corticolimbic circuitry linked to reward (approach) as well as cortico-amygdala circuits linked to threat and fear (avoidance)^8^. Since the neural substrates underlying ‘social’ approach and avoidance are not distinct from those underlying ‘non-social’ approach and avoidance^8–10^, the GAAO posits that the modulatory effects of OT should not be limited to social behaviors^8^. Furthermore, the GAAO also reconciles and extends the social salience account by positing that OT may indeed enhance the attentional salience of many social cues (e.g. as reported in^11^), but not because they are social per se; but because many social stimuli are emotionally evocative and personally relevant^8^. In line with the important role of ‘personal relevance’, the Allostatic theory of OT was recently put forward, providing a theoretical evolutionary-informed framework that OT’s action is centered around the maintenance of stability in shifting environments by flexibly promoting the processing of both social and non-social cues that are relevant for survival^12^.

In a recent study, the effect of OT on behavioral avoidance was investigated using an elegantly designed behavioral paradigm in which participants could reduce the viewing time of neutral and negatively valenced social and non-social stimuli by pressing ‘up’ or ‘down’ on a keyboard^13^. Among participants for whom negative emotion (anxious arousal) is motivationally-relevant, OT was shown to reduce behavioral avoidance of both social and non-social negatively valenced stimuli, providing important evidence that modulations of avoidance-related tendencies by OT are not limited to emotionally-evocative social stimuli, but extend to non-social stimuli as well.

At the neurophysiological level, electroencephalographic (EEG) frontal alpha asymmetry is a widely adopted index for assessing approach/avoidance motivational tendencies^14,15^. Several studies have shown that greater relative left-sided frontal activity is linked to approach motivation, whereas relative right-sided frontal activation reflects withdrawal motivation^16–19^. While OT is postulated to exert its complex effects through a modulation of approach-avoidance tendencies, only one study to date directly assessed the effect of single-dose OT administration on EEG frontal alpha asymmetry. In this prior study, OT was shown to increase approach-related left-sided frontal asymmetry upon engagement of eye contact with a live model, particularly in individuals with lower self-reported social motivation^20^. However, this prior study only assessed EEG frontal alpha asymmetry upon ‘socially’ relevant vs. irrelevant eye contact stimuli. It remains therefore unclear whether the effects of OT on neurophysiological approach-avoidance will also extend toward non-social stimuli, as predicted by the GAAO account.

In the current double-blind, randomized, placebo-controlled study with parallel design, the effect of OT on approach-avoidance-related motivational tendencies was explored both at the behavioral and neurophysiological level in order to experimentally test predictions based on the GAAO account of OT. Before and after nasal spray administration, participants were presented with a series of affective stimuli selected from the Nencki Affective Picture System database (NAPS^21^), depicting social and non-social scenes with either positive (e.g., smiling child, refreshing beverage) or negative valence (e.g., person in despair, dirty toilet). During stimulus presentation, continuous EEG recordings were performed to assess frontal alpha asymmetry, an established neurophysiological index of approach-avoidance motivational tendencies^14,15^. At the same time, participants were able to shorten or lengthen the viewing time of these stimuli by pressing the “up” or “down” key on a keyboard, yielding a behavioral measure of the participants’ approach-avoidance motivational tendencies (i.e. similar key press task (KPT) to the one adopted previously by^13^.

Importantly, before starting the experimental procedure, participants were asked to individually rate perceived valence and sociality of each presented picture, allowing to obtain an assessment of inter-individual differences in self-ratings of the presented pictures, depending on prior person-dependent experiences or personal history. In short, for the ratings of perceived valence, participants were asked to rate the presented pictures on a scale ranging from 1 = very unpleasant (very negative) to 9 = very pleasant (very positive), and ratings were subdivided in four categories yielding for each participant a set of stimuli that was rated *highly* positive or negative (i.e., with high self-rated evocativeness/relevance) and a set of stimuli that was rated only *moderately* positive or negative (low self-rated evocativeness/relevance ). In this way, stimuli factorially varied in terms of ‘affective valence’ (negative vs. positive) and ‘valence intensity’ (high vs. low self-rated evocativeness/relevance). Also for the ratings of sociality, participants were asked to indicate on a 9-point scale ‘how social they rated the presented pictures’, ranging from 1 = very non-social to 9 = very social, and ratings were subdivided in a self-rated ‘social’ and ‘non-social’ category. While compared to valence, ratings of sociality may be less prone to inter-individual variability depending on person-dependent experiences, it can be anticipated that, for example, a picture of a puppy can be rated into the ‘social’ category by a person who owns a dog, while perhaps the same picture may be rated into the ‘non-social’ category by someone who had no experiences with dogs.

Overall, we hypothesized OT to *enhance* approach-related motivational salience, irrespective of sociality (i.e. similar effect towards social and non-social rated stimuli), both behaviorally (KPT) and at the neural level (EEG). Furthermore, we hypothesized the effect of OT on approach motivation to be most pronounced for stimuli with a high level of individually rated evocativeness/relevance (high versus low rated valence intensity), but irrespective of whether they were rated as highly positive or highly negative (affective valence).

## Results

### Effect of OT on approach-avoidance behavior

Figure 2A visualizes pre-to-post changes in behavioral approach-avoidance (key press task, KPT), separately for the OT and PL treatment groups. Mixed-effects analyses on approach-avoidance behavior (with the random factor ‘subject’ and the fixed effect factors ‘treatment group’ (OT vs. PL), ‘sociality’ (social vs. non-social), ‘affective valence’ (negative vs. positive) and ‘valence intensity’ (high vs. low evocativeness/relevance)) revealed a significant ‘treatment x affective valence’ interaction (*F*(1,1805) = 20.05; *p* < 0.001; partial η^2^ = 0.011), indicating a differential OT treatment effect on behavioral approach-avoidance, depending on whether the presented stimuli were self-rated to be negative or positive. Particularly, as visualized in Fig. 2A, the viewing time of the presented stimuli was significantly prolonged after OT for stimuli that were self-rated to have negative valence (OT vs. PL: *F*(1,1805) = 4.23; *p* = 0.04; *d* = 0.687), but not for stimuli that were rated to have positive valence (OT vs. PL: *F*(1,1805) = 0.06; *p* = 0.80; *d* = 0.082). No significant interaction was revealed between treatment and valence intensity (*F*(1,1805) = 0.71; *p* = 0.40) or between treatment and sociality (*F*(1,1805) = 0.05; *p* = 0.82). Also no significant main effect of treatment (*F*(1,50) = 1.18; *p* = 0.28) or ‘treatment x affective valence x sociality’ interaction were revealed (*F*(2,1805) = 1.77; *p* = 0.17).

### Effect of OT on EEG frontal alpha asymmetry

Figure 2B visualizes pre-to-post changes in EEG frontal alpha asymmetry scores separately for the OT and PL treatment groups. Mixed-effects analyses on EEG frontal alpha asymmetry revealed a significant ‘treatment x valence intensity’ interaction (*F*(1,1384) = 7.51; *p* = 0.006; partial η^2^ = 0.005), indicating that the treatment effect of OT on frontal alpha asymmetry was modulated depending on the self-rated valence intensity (high versus low self-rated evocativeness/relevance) of the presented stimuli. As visualized in Fig. 2B, closer examination of the interaction effect showed that—irrespective of affective valence (positive or negative)—approach-related (left-sided) EEG frontal alpha asymmetry was specifically enhanced after OT for stimuli that were self-rated to have high evocativeness/relevance (OT vs. PL: *F*(1,1384) = 5.22; *p* = 0.02; *d* = 0.763), whereas no significant change in EEG frontal alpha asymmetry was evident for stimuli that were rated to have low evocativeness/relevance (OT vs. PL: *F*(1,1384) = 0.05; *p* = 0.48; *d* = 0.075). Accordingly, after OT, stimuli with high evocativeness/relevance elicited stronger approach-related EEG frontal alpha asymmetry, compared to stimuli with low evocativeness/relevance (*F*(1,1384) = 6.65; *p* = 0.01; *d* = 0.84). In the PL group, on the other hand, a significant opposite pattern was evident, indicating a stronger reduction in frontal alpha asymmetry (lower motivational salience) toward stimuli with high evocativeness/relevance (*F*(1,1384) = 4.00; *p* = 0.04; *d* = 0.65). No significant interaction was revealed between treatment and affective valence (*F*(1,1384) = 0.82; *p* = 0.36), or between treatment and sociality (*F*(1,1384) = 0.30; *p* = 0.58). Also no significant main effect of treatment was revealed (*F*(1, 38) = 0.60; *p* = 0.44) or ‘treatment x affective valence x sociality’ interaction were revealed (*F*(2,1805) = 2.05; *p* = 0.13).

### Examining the relationship between OT-induced changes in neural and behavioral approach/avoidance

To understand the relationship between OT-induced changes in neural (EEG frontal alpha asymmetry) and behavioral (changed viewing time during KPT) indices of approach/avoidance, we here performed Spearman correlation analyses exploring whether pre-to-post changes in neural approach were associated with similar pre-to-post changes in behavioral approach (within-subject, across pictures).

For participants of the OT group, Spearman *R* correlation coefficients ranged between *R*_(36)_ = -0.15 and 0.50 (mean 0.044), with two participants showing a significant positive relationship. For participants of the PL group, *R*-coefficients ranged between *R*_(36)_ = -0.24 and 0.40 (mean 0.042), with one participant showing a significant positive relationship. Spearman *R*-coefficients of participants of the OT group were not significantly different from coefficients of the PL group (*t*(38) = -0.027; *p* = 0.97). On average, Spearman *R* coefficients were not significantly different from zero (mean *R* = 0.043; *t*(39) = 1.66; *p* = 0.10). Also between-subject correlation analyses revealed no significant relationship between pre-to-post changes in neural approach and changes in behavioral approach for any of the stimulus types (negative/high evocativeness, negative/low evocativeness; positive/low evocativeness; or positive high evocativeness), either in the OT and PL groups separately, or across groups (all, p > 0.1). Accordingly, while as a group, individuals receiving OT demonstrated higher behavioral and neural approach (particularly upon negatively valenced stimuli), inter-individual differences in the extent of pre-to-post changes in either measure were not explicitly related.

## Discussion

OT was shown to modulate EEG frontal alpha asymmetry, indicating increased approach-related left-sided frontal asymmetry, particularly upon processing stimuli with high self-rated evocativeness/relevance, but irrespective of affective valence (negative or positive) or the social nature of the stimuli (social or non-social). Behavioral approach-avoidance was also modulated by OT, indicating higher approach behavior (sustained relative viewing times) particularly towards negatively valenced stimuli, irrespective of the social nature of the presented stimuli.

Using a similar key press task, Harari-Dahan and Bernstein (2017) recently showed that—for individuals for whom negative emotion is relevant—OT specifically reduced behavioral avoidance towards negatively valenced stimuli, irrespective of whether the stimuli were social or non-social^13^. The current study broadly replicated this pattern of results, showing that—irrespective of whether the stimulus was social or not—approach behavior was higher after OT, compared to placebo, specifically towards negatively valenced stimuli. In terms of the neural assessments of EEG frontal alpha asymmetry, a significant modulation depending on the emotional evocativeness/relevance of the presented stimuli was evident, indicating that OT predominantly enhanced approach-related motivational tendencies for stimuli that were rated to be of high evocativeness/relevance, but irrespective of affective valence (negative or positive) or their social nature. The identification of these stimulus-specific modulations are overall in line with predictions based on the GAAO-account, positing that the modulating effects of OT will specifically include an amplification of approach-related motivational salience towards personally relevant and emotionally evocative cues, irrespective of whether they are social or non-social in nature^8^. Accordingly, processing of both social and non-social cues may be facilitated by OT, not because of their sociality, but because they are emotionally evocative and personally relevant. Note that, related to the design of our study, participants were presented with the same set of pictures for three times (first for the self-rating of the stimuli, next during the KPT before and after nasal spray administration), which may have rendered some habituation to the presented stimuli set. Accordingly, the effects of OT can be interpreted to reflect, at least in part, a modulation of otherwise ‘normal’ habituation effects, seen in the PL group. In line with this interpretation, the PL group mainly displayed a pre-to-post reduction in approach-related motivational salience to relevant stimuli (possibly reflecting habituation), whereas in the OT group, an opposite pattern, reflecting sustained higher motivational salience was evident.

At the behavioral level, a significant modulation depending on affective valence was identified (higher approach upon negative valenced stimuli, compared to positive stimuli), whereas in terms of neural EEG results, a significant modulation depending on the evocativeness/relevance of the presented stimuli was evident (higher approach upon stimuli with high emotional evocativeness, irrespective of affective valence (positive or negative)). Accordingly, only for the negatively valenced stimuli with the highest rated evocativeness, changes in the behavioral and neural measures were evident in a similar direction, indicating a pre-to-post increase in motivational approach (see Fig. 2). For the positive stimuli, a differing pattern of results was revealed, indicating that only for the EEG frontal asymmetry measure, treatment-specific effects of sustained higher neural approach were evident, whereas in terms of behavioral approach (prolongation of stimulus presentation time) both the OT and PL group showed similar increases from the pre to the post session. One interpretation for the differential pattern of results of the behavioral and neural data might be that for eliciting behavioral approach upon positive stimuli, a ceiling effect was at play, such that treatment-specific increments in behavioral approach were masked due to the overall high levels of approach seen both in the OT and PL group (high number of up key presses, prolonging stimulus presentation times). Accordingly, these ceiling effects could have rendered the explicit behavioral task less sensitive for picking up more subtle, implicit changes in approach/avoidance-related motivational salience upon positively valenced stimuli, as identified in the EEG frontal alpha asymmetry data. Nonetheless, observations of stronger OT effects for negative, compared to positively valenced stimuli have been reported before, with prior meta-analytic reports showing that intranasal administration of OT impacts emotion recognition primarily for faces with negative valence (fearful, disgust, anger)^22,23^. Previously, these valence-dependent observations have been interpreted to reflect a modulation of OT’s effects by social boundaries, suggesting that while ‘safe’ stimuli primarily induce pro-socialilty, ‘unsafe’ stimuli primarily induce increased defensiveness, i.e., facilitating increased attention and alertness towards potentially threatening negative social-emotional cues, thereby enhancing their recognition and processing^24^. In line with this notion, the OT group showed sustained higher motivational salience, particularly towards negative stimuli, as indexed with the behavioral KPT task. For the positive stimuli, on the other hand, the effect of OT on sustained higher motivational salience was only evident for the more sensitive neural measure of frontal asymmetry, whereas no treatment-related differences were observed in terms of behavioral approach (both groups displayed comparably high behavioral approach). Indeed, at the neural level, effects were not limited to negative stimuli, but rather modulated depending on the attributed emotional evocativeness/relevance of the stimuli, i.e., irrespective of negative or positive affective valence.

Aside the interpretation of a possible ceiling effect in behavioral approach, the lack of a relationship between the behavioral and neural measures may suggest that the behavioral key press task and EEG frontal asymmetry are not measuring the same kind of motivational mechanism. Indeed, while both are put forward as a proxy for measuring approach/avoidance motivational tendencies, the possibility cannot be ruled out that both measures do not assess the exact same qualitative construct. While the behavioral key press task may assess motivational approach behavior more explicitly, EEG frontal asymmetry is suggested to reflect a more broad state of motivational and/or emotional responding, indicative of enhanced attentional salience attribution, rather than expressing approach-related motivation per se^25^. Especially for EEG frontal asymmetry, modulations depending on personal relevance have been put forward, indicating that motivational direction (high versus low relevance), rather than affective valence (positive versus negative valence) accounts for modulations in frontal cortical activity^26^. Accordingly, also the observed modulations in EEG frontal alpha asymmetry of the current study are interpreted to reflect a role of OT in amplifying attentional salience and preferential processing of environmental cues that are of high personal relevance. This notion aligns well with the recently proposed Allostatic theory of OT, suggesting that OT’s action is centered around the maintenance of stability in shifting environments by flexibly promoting the processing of both social and non-social cues that are relevant for survival (e.g. detection of food, threats, or partners)^12^. Albeit speculative, the observed effects of the current study could therefore point towards a general role of OT in promoting survival-related processes, i.e., by directing more attentional and motivational resources to the evaluation of significant environmental cues.

Previously, a role of OT in amplifying signal value/salience by enhancing signal-to-noise ratios of neuronal transmissions has been proposed^27,28^. In vivo rodent research has demonstrated that OT-receptor activity increases peak firing responses to social sensory information (odorants) at the level of mitral neurons in the olfactory bulb, by lowering their (spontaneous) baseline firing, and hence augmenting the signal-to-noise ratio for signal transmission^27^. Similar actions of OT on neural coding were observed in rodent hippocampal recordings, indicating that OT specifically enhanced the signal-to-noise ratio in neuronal spike transmission^28^. Accordingly, the induction of high signal-to-noise states has been suggested to form a shared over-arching mechanism by which OT may amplify signal value of neuronal transmissions within wide-spread neural circuits^28^. In translating these neuronal findings to a brain’s system level, it is speculated that OT may exert a similar role in promoting high signal-to-noise states, i.e., by amplifying the signal-value of highly salient (e.g., survival-relevant) sensory input, while lowering the allocation of neural resources to the processing of other (background) environmental cues, irrelevant to the organism’s survival (reducing noise). In other words, OT may act as a neuromodulatory filter, promoting the extraction of survival-relevant information from other, more irrelevant background stimuli (noise). The current observation that after OT, stimuli with high emotional evocativeness elicited stronger left-sided EEG frontal alpha asymmetry (indicative of approach motivational tendencies), compared to stimuli with low emotional evocativeness, provides support to the notion that OT may enhance the differential processing of stimuli with high versus low personal relevance.

In humans, intranasal administration of OT is increasingly considered as a potential treatment for several neuropsychiatric conditions with deficits in the social domain, including ASD^29^. Although poorly understood, aberrant sensory processing forms a core aspect of ASD symptomatology^30^, typically characterized by either exaggerated (hyper) or diminished (hypo) reactivity to sensory input, indicating an imbalance in filtering sensory information from the external surroundings^31^. Albeit speculative, OT’s suggested role in promoting attentional salience to signal input with high versus low relevance may provide an entry point for balancing exaggerated responses or hypersensitivity to (background) noise.

While the observed effects and suggested implications are of relevance, the inclusion of only a limited sample of healthy adult men is noted as a limitation. Future trials will be needed to explore whether the observed effects will generalize in larger, more representative samples, additionally including women as well as clinical populations (e.g., ASD) for which OT is increasingly considered as a potential treatment^32^.

To conclude, administration of OT was shown to modulate approach-avoidance tendencies at the behavioral and neural level, irrespective of the social nature of the presented stimuli. At the neural level, OT specifically amplified the approach-related motivational salience of stimuli with high personal relevance. Together, these findings suggest that—irrespective of stimulus’ valence or social/non-social nature—OT will amplify the motivational salience of environmental stimuli, given that they are of high (personal) relevance.

## Methods

### Participants

Participants were recruited to participate in a single lab-session during which neurophysiological recordings (EEG) were performed during a key press task (KPT), before and after nasal spray administration of oxytocin (OT) or placebo (PL). A total of 52 (male) participants completed the experimental recordings, of which 26 received the OT nasal spray (mean age 22.73 ± 3.04 years) and 26 received the PL nasal spray (mean age 22.19 ± 2.40 years). Quality control check of the recorded EEG data showed that data of 12 participants could not be included due to missing signal or excessive noise in one of the mastoid reference electrodes (1 OT/3 PL) or target frontal electrodes (F3/F4; 3 OT/5 PL). This drop-out was accounted for in the overall recruitment, hence retaining a final sample size of qualitative EEG data for 40 participants (22 OT (mean age 22.50 ± 3.11 years)/18 PL (mean age 22.17 ± 2.53 years)). Note that this is similar to a prior EEG study examining single-dose OT effects on frontal alpha asymmetry^20^.

Inclusion criteria for participation were: male; aged between 18–35 years; native-Dutch speaking; right-handed and corrected-to-normal vision. Only male participants were included to avoid the potential confound of gender-dependent effects of OT^33^. Exclusion criteria encompassed any self-reported usage of psychotropic medication or known neurological or psychiatric condition. Before testing, participants abstained from alcohol and caffeine 24-h prior to testing. Written informed consent was obtained from all participants prior to the study. Consent forms and study design were approved by the UZ/KU Leuven Ethics Committee for Biomedical Research in accordance to The Code of Ethics of the World Medical Association^34^. Participants were recruited to participate in a larger study including assessments of photoplethysmography and electrodermal recordings (not part of the current report). The study design was registered at ClinicalTrials.gov (NCT04443647; 23/06/2020; https://clinicaltrials.gov/ct2/show/NCT04443647).

### Experimental procedure and stimuli

#### Stimuli

**Figure 1:**
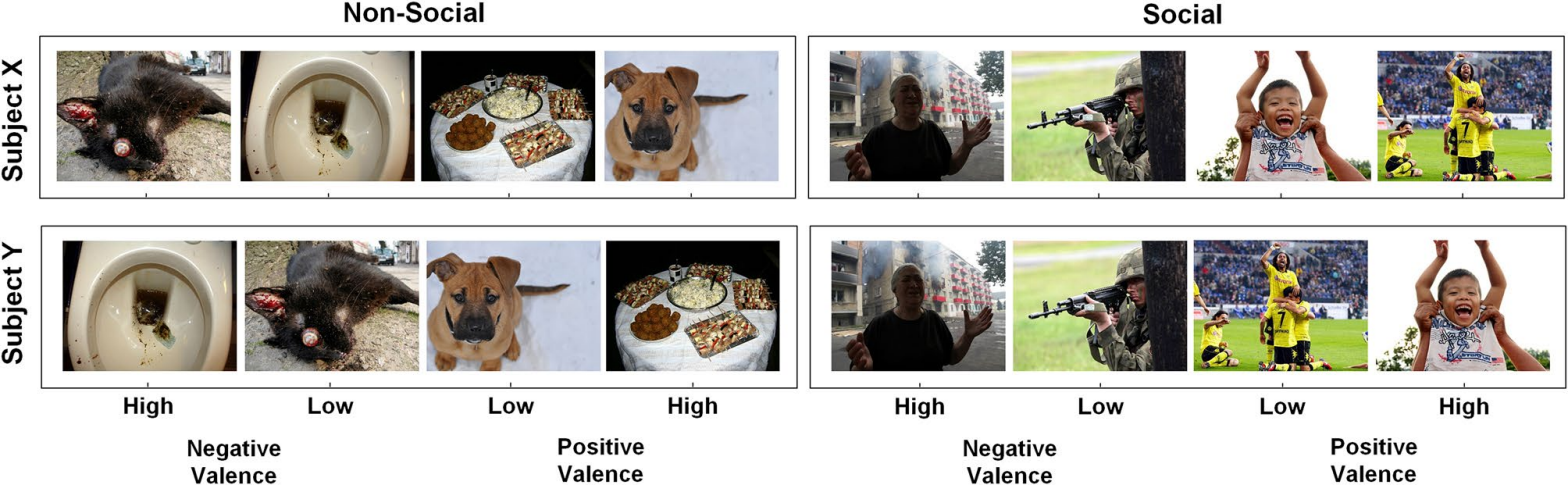
Example of differential picture ratings among two exemplary subjects. Participants individually rated the ‘sociality’ and ‘valence’ of a set of 36 stimulus pictures (here, only 8 exemplary pictures are shown; see Supplementary Fig. 1 for the full stimulus set) to obtain a person-dependent scoring of the perceived social/non-social nature of the presented stimuli, as well as the stimuli’s perceived emotional evocativeness (high, low) in terms of positive/negative valence. For example, a picture of a winning football team can be anticipated to be rated with high positive valence by a person who loves sports (exemplary subject X), whereas the same picture may be rated more neutral (low positive valence) by another subject (exemplary subject Y). Likewise, for a person who owns a cat, a picture of a dead cat may evoke stronger negative valence than the picture of the dirty toilet (exemplary subject X), whereas for others, this may be the other way around (exemplary subject Y). All pictures were selected from the publicly available Nencki Affective Picture System database (NAPS^21^).

Participants were seated in front of a computer screen and were instructed to pay close attention to the presented stimuli. Stimuli comprised 36 pictures from the Nencki Affective Picture System database (NAPS;^21^, selected to represent social and non-social emotionally-evocative stimuli, with either positive (e.g., smiling child, festive buffet) or negative valence (e.g., person in despair, dirty toilet). See Fig. 1 for some exemplary pictures and Supplementary Fig. 1 for the full stimulus set.

#### Subjective rating

At the start of the experimental session (before EEG and key press task recordings), all pictures were presented to the participants for 3 s in a semi-random order (no more than two consecutive stimuli of the same picture category) and participants were asked to individually rate the ‘valence’ (‘*How pleasant do you rate the following pictures?*’) and ‘sociality’ (*‘How social do you rate the following pictures?’*) after presentation of each picture using a 9-point visual analog scale (i.e., 1 = very unpleasant/non-social, 9 = very pleasant/social). Generally, participants were asked to ‘try to rate the pictures according to their own experience/interpretation’ and it was noted that ‘there are no right or wrong answers’. The subjective ratings allowed obtaining for each participant: (1) a person-dependent scoring of the perceived social nature of the presented stimuli, as well as (2) the stimuli’s perceived emotional evocativeness in terms of negative/positive valence. Ratings of ‘sociality’ of the presented stimuli were subjected to a median split, yielding for each participant a self-rated ‘social’ and ‘non-social’ category. Self-ratings of valence were subjected to a quartile split, yielding for each participant a set of negative and positive stimuli with high versus low emotional evocativeness, i.e., varying according to ‘affective valence’ (negative vs. positive) and ‘valence intensity’ (high vs. low evocativeness). Figure 1 visualizes an example of differential picture ratings among two exemplary subjects.

#### Key press task (KPT)

After the subjective rating, continual neurophysiological recordings were obtained while participants performed a KPT, before (baseline) and (30 min) after (post) nasal spray administration. The stimulus set of pictures was presented in a semi-random order and participants were informed that they could either prolong or shorten the viewing time of each presented stimulus by pressing the ‘up’ or ‘down’ arrow key on a keyboard, respectively. The viewing time was adjusted in real-time according to the formula: New Total Time = Old Total Time + (Extreme Time – Old Total Time)/K. The Extreme Time was set to 12 s for extending the viewing time and to 0 s for shortening the viewing time; K was a scaling constant set to 40 (similar paradigm as adopted in^13^ and based on^35^). Accordingly, an up or down key press prolongs or shortens the viewing time with respectively, 0.15 s (firs key press); 0.146 (second key press), 0.142 (third key press); 0.139 (fourth key press); etc. If participants did not press any arrow key, the stimulus presentation was discontinued after 6 s. In order to avoid the possibility that participants would shorten all trials in order to complete the experimental session more quickly, participants were told that the total duration of the task was fixed. For each picture, a behavioural measure of approach/avoidance was obtained by assessing the total sum of ‘up’ (positive value) and ‘down’ (negative value) key presses. Accordingly, a positive total sum-score indicates a higher number of ‘up’ key presses (i.e., prolonging the viewing time, indicative of approach) and a negative total sum score, indicates a higher number of ‘down’ key presses (i.e., shortening the viewing time, indicative of avoidance). The average total sum score (across participants and stimuli) was 0.8 ± 11.06 (mean ± SD), meaning that, on average, participants prolonged the stimulus viewing time with one ‘up’ key press, to an average picture viewing time of 6.15 s.

#### Materials and apparatus

E-prime 3® software (Psychology Software Tools, USA) was used for stimulus presentation and response recording during the KPT. The NeXus-32 multimodal acquisition system and corresponding BioTrace + software (version 2018a, Mind Media, The Netherlands) were used to collect neurophysiological recordings (synchronized to E-prime using the Nexus Trigger Interface) (similar to^20^). Overall, procedures for EEG recordings and data handling were similar to those described in^20^.

#### EEG recordings and data handling

Continuous EEG was recorded during the KPT task (before (baseline) and after (post) nasal spray administration), with a 21-electrode cap (MediFactory, The Netherlands) positioned according to the 10–20 system. Skin abrasion and electrode paste (NuPrep) were used to reduce electrode impedance. Electro-oculography (EOG) was performed to record vertical (vEOG) and horizontal (hEOG) eye movements by placing pre-gelled foam electrodes (Kendall, Germany) above and below the left eye and next to the left and right eye. The sampling rate of the EOG recordings was 2048 Hz. The EEG-signal was amplified using a unipolar amplifier and referenced offline to linked mastoids (electrodes A1 and A2). The sampling rate for the digitized EEG-signal was 256 Hz.

EEG data were analyzed using BrainVision Analyzer 2.2 software (Brain Products GmbH, Germany). The EEG-signal was filtered using a 0.5 Hz low cut-off, a 70 Hz high cut-off and a 50 Hz notch filter. After visual inspection, deflections resulting from eye blinks and horizontal eye movements (assessed using the vEOG- and hEOG-channels), were removed by the implemented Independent Component Analysis (ICA) Ocular Correction module in BrainVision Analyzer 2.2^36^. Thereafter, each trial was segmented into epochs of 1 s with an overlap of 0.5 s, resulting in 9 epochs for each of the 36 affective stimuli. For consistency, a trial length of 5 s was adopted, ensuring a fixed number of included time points for each stimulus. This trial duration was chosen to maximize the amount of included time points, still ensuring for > 85% of the trials, that the trial duration fully overlapped with the picture presentation time (i.e., on average, the stimulus presentation constituted 6.15 s).

Epochs with residual artifacts exceeding ± 100 µV in amplitude were rejected. For each presented stimulus, averaged power spectra were calculated over epochs using BrainVision-implemented Fast Fourier transformation (FFT) and power spectral density (µV^2^/Hz) within the alpha-band (8–13 Hz) was computed and ln-transformed. Frontal electrode pairs F3/F4 were used to calculate the frontal alpha asymmetry score, by subtracting the ln-transformed alpha-power values of the left hemisphere (F3) from that of the right hemisphere (F4); [ln(alpha-power F4) – ln(alpha-power F3)]^37^. Since alpha-band power density is inversely related to cortical activity^38^, a positive asymmetry value indexes stronger relative left-sided frontal brain activity (lower frontal alpha in left F3, compared to right F4) associated with approach motivation, whereas a negative value indexes stronger relative right-sided frontal brain activity (lower frontal alpha in right F4, compared to left F3) associated with avoidance motivation.

### Nasal spray administration

Participants were randomly assigned to receive a single-dose of OT or PL based on a computer-generated randomized order. Except for the manager of randomization and masking of drug administration, all participants and research staff conducting the study were blind to treatment allocation. In correspondence with previous studies^39^, participants received 24 international units (IU) of OT (Syntocinon®, Sigma-tau) or PL containing a saline natriumchloride solution. Participants received 3 puffs/nostril in an alternating fashion with each puff containing 4 IU. For inhalation of the spray, participants were instructed to take a deep breath through the nose and to tilt their head slightly backwards during nasal administration in order to minimize gravitational loss of the spray (according to recommendations for standardized use^40^.

In healthy humans, the impact of OT on social cognition is commonly evaluated using a 30–45 min wait-time before initiation of the experimental task (^39^. Accordingly, in the current study, a 30-min wait-time was incorporated prior to initiating the post-session recording in order to assess the effects of OT during peak OT-concentrations. All participants were monitored for potential side effects until approximately one-hour after nasal spray administration. Only minimal and non-treatment specific side effects were reported, although note that a slightly larger number of participants receiving the OT spray reported watery eyes (4 in OT, 0 in PL group; see Supplementary Table 1). Finally, at the end of the experimental session, participants were asked if they thought they had received the OT or PL nasal spray. The proportion of participants that believed they had received the OT treatment was not significantly larger in the actual OT group (26.56%), compared to the PL group (18.75%) (Pearson Chi-square = 0.06; *p* = 0.80).

### Statistical analysis

**Figure 2:**
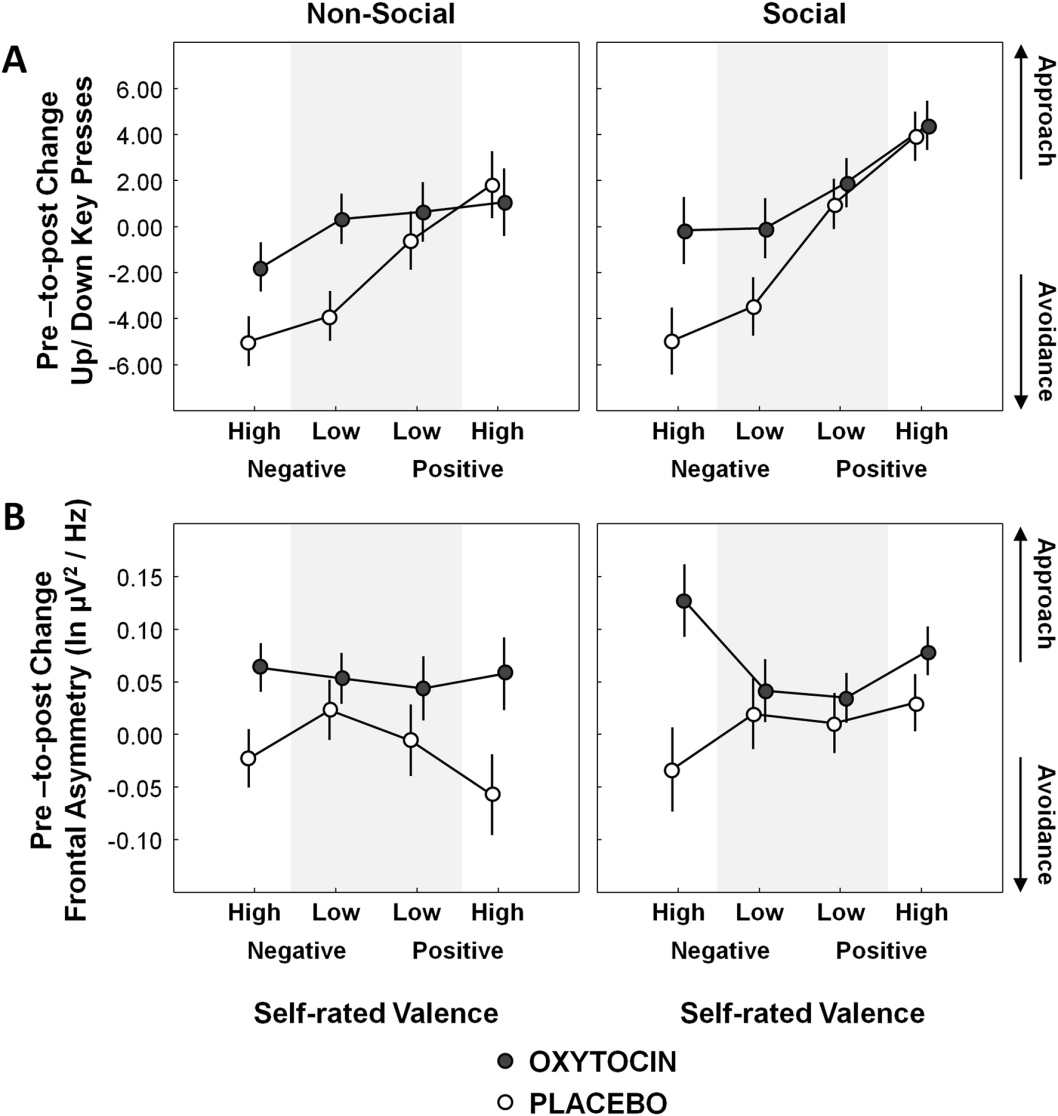
Effect of oxytocin on approach/avoidance as a function of self-rated stimulus characteristics. (**A**) visualizes pre-to-post changes in behavioral approach-avoidance (key press task, KPT) with positive scores indexing increased approach (prolongation of viewing time by pressing the ‘up’ key) and negative scores indexing increased avoidance (shortening of viewing time by pressing the ‘down’ key). (**B**) visualizes pre-to-post changes in EEG frontal alpha asymmetry scores, with positive scores indicating an increase in approach-related left-sided frontal alpha asymmetry. Pre-to-post change scores are visualized separately for each treatment group (oxytocin, placebo), as a function of self-rated sociality (social, non-social), affective valence (negative, positive) and valence intensity (high, low evocativeness). Vertical bars denote  ± standard errors.

To assess treatment-related effects, behavioral (KPT) and neural (EEG-based frontal alpha asymmetry) indices of approach-avoidance motivation recorded post nasal spray administration (separately for each stimulus) were subjected to mixed-effects analyses of variances (ANOVAs). The factor ‘subject’ was inserted as a random effect, whereas the factors ‘treatment group’ (OT vs. PL), ‘sociality’ (social vs. non-social), ‘affective valence’ (negative vs. positive) and ‘valence intensity’ (high vs. low evocativeness); as well as all interactions with the factor ‘treatment group’, were inserted as fixed effects. To correct for a potential effect of individuals’ baseline scores, baseline values prior to nasal administration were included as covariates in the models. Accordingly, results figures visualize pre-to-post change scores, with positive values indicating increased approach post-treatment (Fig. 2). All statistics were executed with Statistica version 13 (TIBCO Software Inc., USA). Results were considered significant when p < 0.05. Cohen’s d effect sizes of between-group differences are reported, where 0.2 is indicative of a small effect, 0.5 a medium effect and 0.8 a large effect.

## Supplementary Information

Supplementary Information.
