# Supplementary material for: Oxytocin enhances neural approach towards social and non-social stimuli of high personal relevance
Source: Sci Rep. 2021 Dec 8;11:23589. doi: 10.1038/s41598-021-02914-8 (PMC8655079; doi:10.1038/s41598-021-02914-8)
Supplement: Supplementary file 1 — Supplementary Information. [file 41598_2021_2914_MOESM1_ESM.pdf]

Supplementary Information

Oxytocin enhances neural approach towards social and non-social stimuli of high personal relevance

Authors and affiliations

Kaat Alaerts, Aymara Taillieu, Nicky Daniels, Javier R. Soriano, Jellina Prinsen

Supplementary Figure 1

Example pictures of adopted affective stimuli. A total of 36 pictures were selected from the publicly available Nencki Affective Picture System (NAPS) database (Marchewka et al., 2014), representing social versus non-social emotionally-evocative stimuli, with either positive or negative valence. Social stimuli were a priori selected to contain people, human body parts (e.g. faces) or social scenarios. A priori-selected categories of social and non-social pictures were matched on valence, with a mean rated valence (included in the NAPS database) of 7.64 ± 0.13 SD (social pictures, *n* = 9) and 7.73 ± 0.24 (non-social pictures, *n* = 9) for the positively valenced pictures and a mean rated valence of 2.65 ± 0.58 (social pictures, *n* = 9) and 2.50 ± 0.60 (non-social pictures, *n* = 9) for the negatively valenced pictures.

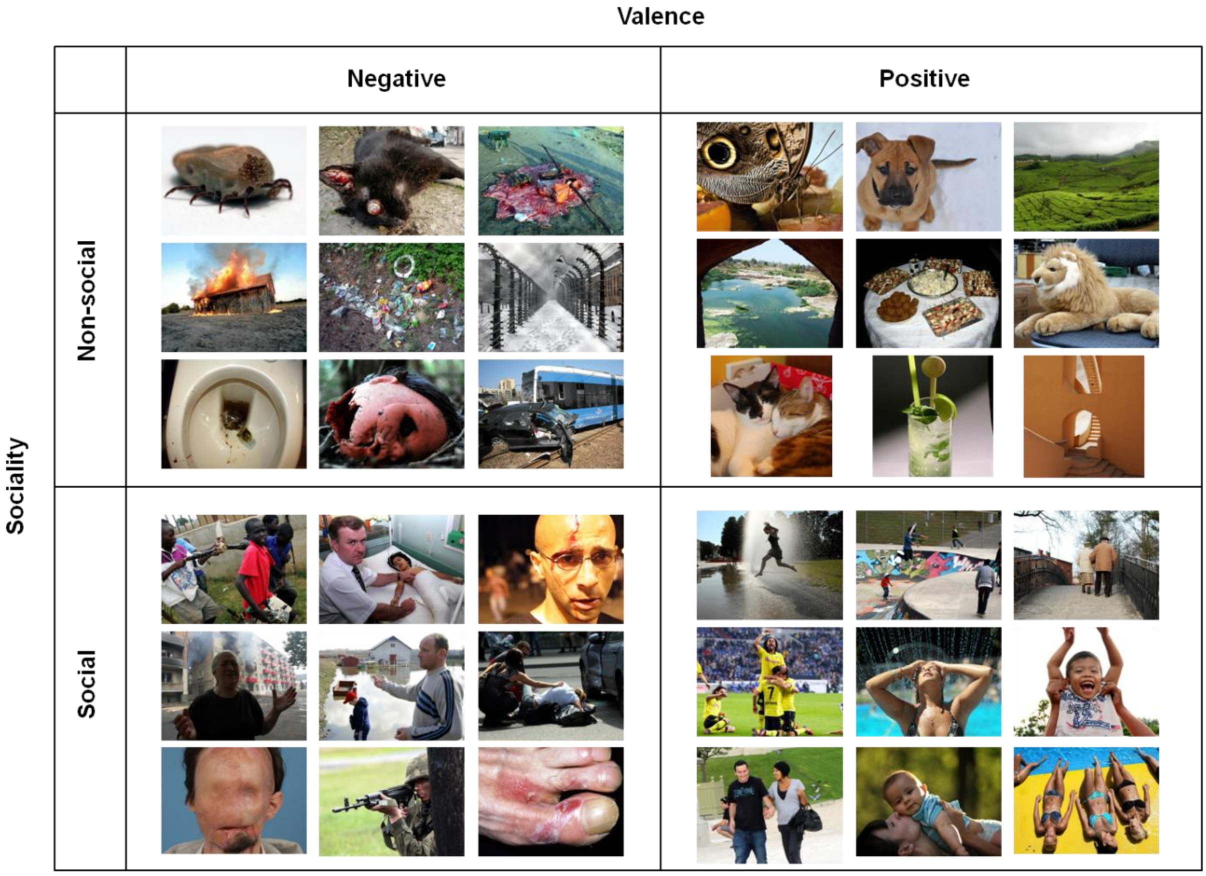

Supplementary Table 1

Side effects questionnaire. At the end of the experimental session, participants were asked to report whether they presented any of the listed (or other) side effects and to indicate the severity of the side effect (mild, moderate, or severe). The number of oxytocin (OT) participants or placebo (PL) participants that reported any mild, moderate or severe side effects are listed separately for each side effect. A significant group difference (Pearson Chi-square test, * $p < .05$) was noted for the side effect 'watery eyes', indicating that a larger number of participants in the OT group experienced mild ($n = 3$) to moderate ($n = 1$) watery eyes.

**Side effect**	Mild		Moderate		Severe		Total			
	OT	PL	OT	PL	OT	PL	OT	PL	Chi-square	p-value
Headache	0	2	2	0	0	0	2	2	0.00	1.00
Drowsiness	9	10	5	6	1	0	15	16	0.63	0.80
Dizziness	0	1	1	0	0	0	1	1	0.00	1.00
Fainting	0	0	0	0	0	0	0	0	0.00	1.00
Changes in heart rate or palpitations	2	1	0	0	0	0	2	1	0.35	0.55
Shortness of breath	1	1	0	0	0	0	1	1	0.00	1.00
Fever	0	0	0	0	0	0	0	0	0.00	1.00
Sore throat	0	0	0	0	0	0	0	0	0.00	1.00
Dry throat/dry mouth	4	5	0	2	0	0	4	7	0.99	0.32
Hoarseness	0	0	1	1	0	0	1	1	0.00	1.00
Coughing	0	1	0	0	0	0	0	0	1.02	0.31
Coughing up mucus	1	1	0	0	0	0	1	0	0.00	1.00
Congested nose	1	2	0	1	0	0	1	2	1.07	0.30
Sneezing	0	0	0	1	0	0	0	0	1.02	0.31
Nasal irritation	1	2	0	1	0	0	1	2	1.07	0.30
Runny nose	3	2	1	0	0	0	4	2	0.74	0.39
Watery eyes	3	0	1	0	0	0	4	0	**4.27**	**0.04***
Nausea and/or vomiting	1	0	0	0	0	0	1	0	1.02	0.31
Abdominal or stomach pain	0	1	0	0	0	0	0	1	1.02	0.31
Changes in perception of the tongue	0	0	0	0	0	0	0	0	0.00	1.00
Burning sensation in nose and/or ears	0	1	0	0	0	0	0	1	1.02	0.31
Muscle pain/cramps	0	0	0	0	0	0	0	0	0.00	1.00
Skin rash	0	0	0	0	0	0	0	0	0.00	1.00
Sweating	1	0	0	0	0	0	1	0	1.02	0.31
Sensitive to fragrances	0	0	0	0	0	0	0	0	0.00	1.00
Blurred vision	1	2	0	0	0	0	1	2	0.35	0.55

References

Marchewka A, Zurawski Ł, Jednoróg K, Grabowska A. The Nencki Affective Picture System (NAPS): introduction to a novel, standardized, wide-range, high-quality, realistic picture database. *Behav Res Methods* **46**, 596-610 (2014).
